# Spontaneous adaptation explains why people act faster when being imitated

**DOI:** 10.3758/s13423-016-1141-3

**Published:** 2016-08-16

**Authors:** Jarosław R. Lelonkiewicz, Chiara Gambi

**Affiliations:** Department of Psychology, University of Edinburgh, 7 George Square, Edinburgh, EH8 9JZ UK

**Keywords:** Joint action, Adaptation, Anticipation, Imitation, Action effects

## Abstract

The human ability to perform joint actions is often attributed to high-level cognitive processes. For example, the finding that action leaders act faster when imitated by their partners has been interpreted as evidence for anticipation of the other’s actions (Pfister, Dignath, Hommel, & Kunde, 2013). In two experiments, we showed that a low-level mechanism can account for this finding. Action leaders were faster when imitated than when counterimitated, but only if they could observe their partner’s actions (Exp. 1). Crucially, when due to our manipulation the partner’s imitative actions became slower than the counterimitative actions, leaders also became slower when they were imitated, and faster when counterimitated (Exp. 2). Our results suggest that spontaneous temporal adaptation is a key mechanism in joint action tasks. We argue for a reconsideration of other phenomena that have traditionally been attributed solely to high-level processes.

**Electronic supplementary material:**

The online version of this article (doi:10.3758/s13423-016-1141-3) contains supplementary material, which is available to authorized users.

When people engage in a joint activity, they tend to closely coordinate their actions. For example, a couple enjoying a night stroll on the beach might walk in synchrony, holding hands and jointly navigating to avoid puddles of water. This could be viewed as a case of planned coordination—one that results from shared representations of the desired outcome and the actions necessary to achieve it. However, coordination can also emerge spontaneously, independent of complex representations and high-level cognitive processes (Knoblich, Butterfill, & Sebanz, 2011). For example, the couple on the beach might coordinate their footsteps as a result of low-level automatic mechanisms present in both agents. In this article, we focus on one such mechanism—that is, temporal adaptation. We propose that spontaneous temporal adaptation can account for some findings that have previously been taken as evidence that agents represent and anticipate each other’s actions.

A great deal of research supports the notion that agents successfully coordinate their actions via high-level processes. For instance, people acting together form and pursue joint goals (Loehr & Vesper, 2016), are aware of each other’s focuses of attention (Böckler, Knoblich, & Sebanz, 2012), mentalize about their coactors’ perspectives (Ryskin, Benjamin, Tullis, & Brown-Schmidt, 2015) and beliefs (van der Wel, Sebanz, & Knoblich, 2014), and form precise representations of each other’s actions (Sebanz, Bekkering, & Knoblich, 2006) and their anticipated outcomes (Pfister, Dolk, Prinz, & Kunde, 2014).

However, there is also clear evidence that people coordinate by engaging simpler mechanisms. Temporal adaptation is a low-level mechanism that is particularly important for interpersonal coordination (Konvalinka, Vuust, Roepstorff, & Frith, 2010) and has been shown to often occur automatically (Keller, 2008; Mills, van der Steen, Schultz, & Keller, 2015). Many forms of human interaction are shaped by the tendency to adapt to each other’s actions. For example, musicians playing a duet adjust their subsequent performance to correct for asynchronies (Goebl & Palmer, 2009), audiences fall into one clapping rhythm (Neda, Ravasz, Brechte, Vicsek, & Barabasi, 2000), interlocutors align on patterns of body sway (Fowler, Richardson, Marsh, & Shockley, 2008), and people rocking in rocking chairs spontaneously synchronize the frequencies of their movements (Richardson, Marsh, Isenhower, Goodman, & Schmidt, 2007).

We believe that adopting a more low-level perspective can inform the efforts to create a comprehensive framework of joint action. Importantly, it can help to address the criticisms that have been proposed against some of the high-level accounts. For instance, mental inferences (Shintel & Keysar, 2009) and anticipation (Pfister, Pfeuffer, & Kunde, 2014) are time-consuming and effortful in terms of cognitive resources, suggesting that their widespread use is unlikely. Furthermore, it has been argued that some aspects of joint action (e.g., synchronization in time) are best explained via low-level mechanisms rather than via common coding and other representational theories (Schmidt, Fitzpatrick, Caron, & Mergeche, 2011). Most importantly, recent studies have suggested that phenomena that have traditionally been interpreted in terms of complex, high-level processes can in fact be explained by much simpler mechanisms (see Dolk et al., 2014, for a review). Here we show that the apparent effect of anticipation of a coactor’s action on one’s own action is one such phenomenon.

Anticipation has been advanced as being key for successful coordination between agents (Knoblich & Jordan, 2003; Kourtis, Sebanz, & Knoblich, 2013). It has been suggested that anticipating the sensory consequences of one’s own action can activate the motor program that normally produces this action (Hommel, 2013; Hommel, Müsseler, Aschersleben, & Prinz, 2001). For example, in one classic study the button presses of a participant were followed by a light effect whose location was either compatible or incompatible with the location of the presses. Actions were initiated faster in the compatible effect condition, suggesting that participants anticipated the location of the effect and used it as a cue to activate the motor program for a spatially corresponding press (Kunde, 2001). In joint action, anticipating the partner’s response could cue the agent to activate the action that typically causes this response (Müller, 2015; Pfister, Dolk, Prinz, & Kunde, 2014). Such anticipation could prime the execution of complementary movements, ultimately benefiting any activity that requires two people to coordinate their actions.

One striking demonstration of this process has come from a recent study by Pfister, Dignath, Hommel, and Kunde (2013). In this study, one participant acted as a leader and performed a short or a long button press in response to a cue on the computer screen. Her partner acted as a follower and was instructed to perform either the same (imitation) or the opposite (counterimitation) type of press. The study showed that the leader initiated her actions faster when she was imitated. The authors interpreted this as evidence for anticipation of the follower’s movements, in line with the literature on compatibility effects and the ideomotor theory (Hommel et al., 2001). However, the follower’s actions were not just compatible in the imitation condition and incompatible in the counterimitation condition; the authors also reported that the follower was faster in the former than in the latter condition. In fact, a large body of research has shown that action execution is facilitated for imitative movements (see Heyes, 2011, for a review).

We propose that a much simpler temporal-adaptation mechanism can account for this finding: The leader adapted her response speed to the follower’s—that is, speeding up when her partner performed the faster imitative movements, and slowing down when he performed the slower counterimitative movements. Although most of the evidence for temporal adaptation has come from research on rhythmic, continuous movements (e.g., Repp, 2005; Repp & Su, 2013), some studies suggest it can also occur in nonrhythmic, discrete tasks (Jung, Holländer, Müller, & Prinz, 2011). We hypothesized that temporal adaptation may play a key role in our task, although this task has previously been used to investigate high-level processes.

We investigated this hypothesis in two experiments. In Experiment 1, we cancelled out visual and auditory feedback about the followers’ performance. We hypothesized that if the effect on leaders’ response times (RTs) is due to temporal adaptation, it should disappear after removing perceptual information about the follower’s behavior. In Experiment 2, we manipulated followers’ responses to elicit a reversed RT pattern—that is, faster in counterimitation, slower in imitation. If leaders were to accommodate their response speed to this atypical pattern, this would be strong evidence in favor of the adaptation hypothesis.

## Experiment 1

### Methods

We invited 24 previously unacquainted participants (20 female, 4 male; all right-handed) to form same-gender pairs. This sample size was chosen on the basis of Pfister et al. (2013). The participants were Edinburgh University students with no reported motor disorders and were paid £6 for their time. The study was approved by the Psychology Research Ethics Committee at Edinburgh University, and informed consent was obtained from all participants.

Participants were randomly assigned the roles of leader and follower and were seated across a table. In each trial, the leader watched the computer screen change color from black to either red or green, indicating a short (1–150 ms) or a long (200–600 ms) response. The color–response mapping was counterbalanced between pairs. The follower was instructed to observe the leader’s action and to perform the same (compatible; imitation condition) or the opposite (incompatible; counterimitation condition) type of button press. After the follower’s response the screen turned black for 1,000 ms, and then the next trial started. The participants were instructed to perform their actions as quickly as possible. The total duration of a trial was 4,000 ms: initial black screen (500 ms) + color cue (2,500 ms) + end trial black screen (1,000 ms).

We presented 14 practice trials at the beginning of each session to familiarize participants with the two press types. After practice, the participants completed one imitation and one counterimitation block. Then they switched roles and completed two more blocks, so that each person completed the task both as leader and as follower. Each block consisted of 120 trials, and the order of blocks was counterbalanced between pairs.

**Fig. 1:**
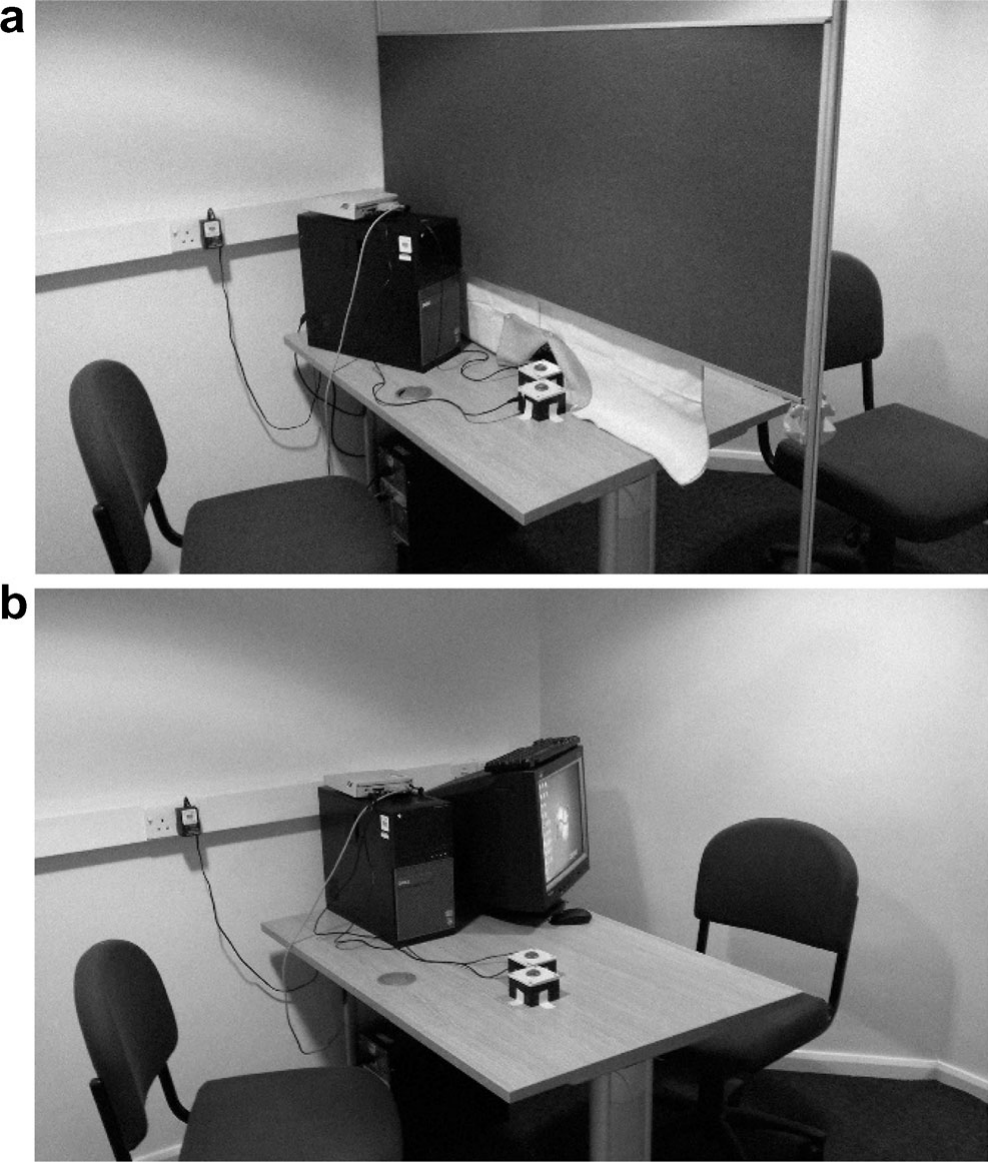
The set-up of Experiments 1 and 2. Panel *a* shows Experiment 1: p The leader is seated on the right-hand side of the divider. The diagonal positioning of the button boxes allows the follower to see both his and the leader’s hands, while the leader can see only her own hand. Panel *b* shows Experiment 2: The participants can freely observe each other

To remove visual and auditory feedback about the follower’s performance, we placed a divider between participants. This setup allowed the follower to see the leader’s hand and the button box, while the leader could not see the follower at all (Fig. 1, panel a). Furthermore, the leader wore earplugs, as well as noise-cancelling headphones (Sony MDR-NC60). To make sure that participants knew what type of response would be performed by their partner in each condition, the instructions were carefully explained to them at the beginning of the session and then repeated before the start of each block (i.e., leaders were told the color–response mapping, informed whether it was an imitation or counterimitation block, and asked to explain how the follower would respond to their actions). At the end of the session, participants were paid and debriefed.

### Results

Following Pfister et al. (2013), we discarded the first 24 trials from each block (warm-up trials). Prior to the analyses of the leader’s responses, we excluded all trials in which the leader performed the wrong type of press (3.03 %). We also excluded outliers deviating more than 2.5 *SD*s from each participant’s condition mean (2.02 %). For the follower’s analyses, we excluded trials in which either participant made an error (8.87 %), and further trimmed the data to remove the follower’s outliers (1.91 %).^1^ Below we focus on the effect of imitation versus counterimitation, both overall and separately for each type of leader’s press; see the supplemental material for the full analysis of variance (ANOVA) results.

We replicated the well-established imitation facilitation effect: Followers’ RTs were shorter in the imitation than in the counterimitation condition (*M* = 318 vs. 459 ms), *t*(23) = 5.79, *p* < .001, 95 % confidence interval (CI) = [92, 195] (all reported *t* tests are two-tailed). To check whether this effect was independent of the leader’s response type, we ran follow-up pairwise comparisons for short and long leader presses (all subsequent analyses refer to the *leader’s* press type). We observed a significant difference (Bonferroni *p* = .025) between the imitation and counterimitation conditions for both long presses (*M* = 308 vs. 392 ms), *t*(23) = 2.82, *p* = .010, *r* = .51, 95 % CI = [24, 154], and short presses (*M* = 328 vs. 526 ms), *t*(23) = 7.64, *p* < .001, *r* = .85, 95 % CI = [148, 258]. This shows that in imitation followers initiated their actions faster, regardless of the type of response performed by the leaders.

**Fig. 2:**
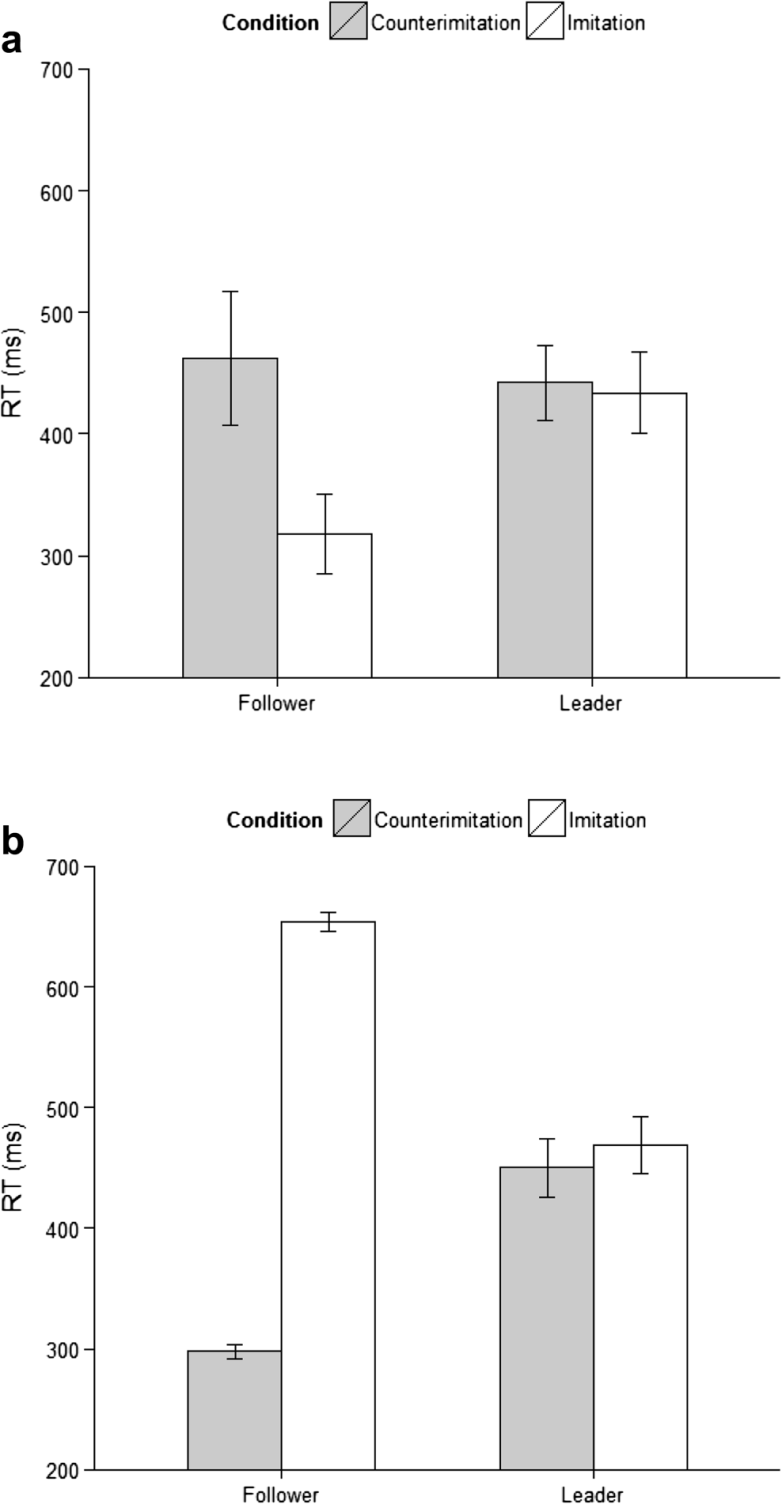
Mean leaders’ and followers’ response times (RTs) in imitation and counterimitation. Error bars represent 95 % confidence intervals. Panel a shows results from Experiment 1, panel b results from Experiment 2

Importantly, however, leaders were not faster when they were imitated than when they were counterimitated, suggesting that removing perceptual feedback considerably attenuated any influence of the partner’s performance (*M* = 434 vs. 441 ms), *t*(23) = 1.20, *p* = .241, *r* = .24, 95 % CI = [–5, 19] (Fig. 2, panel a). Follow-up pairwise comparisons showed no difference between imitation and counterimitation for long presses (*M* = 444 vs. 443 ms), *t*(23) = –0.15, *p* > .250, *r* = .03, 95 % CI = [–16, 13]. For short presses, the *p* value for the condition effect was just below the conventional alpha threshold, but not below the threshold after correction for multiple comparisons (*M* = 425 vs. 440 ms), *t*(23) = 2.10, *p* = .047, *r* = .40, 95 % CI = [0, 29] (Bonferroni *p* = .025).

### Discussion

Our results suggest that the effect on leaders’ RTs reported by Pfister et al. (2013) was not due to anticipation. If it were, we should have observed a reliable difference between the conditions even when leaders were unable to observe the followers’ actions. There is evidence that coactors seated in separate rooms can represent each other’s actions (Atmaca, Sebanz, & Knoblich, 2011; Gambi, Van de Cavey, & Pickering, 2015). Therefore, a strong version of the anticipation account would predict that merely knowing whether the partner would respond with a compatible or an incompatible action should influence the leader’s action execution (Pfister, Pfeuffer, & Kunde, 2014).

However, it is possible that by cancelling perceptual feedback we made it impossible for the leader to represent the follower’s response as a consequence of their action. Under a weaker version of the anticipation account, leaders integrate the followers’ actions into representations of the outcomes of their own actions only if they can directly observe the followers. Therefore, in Experiment 2 we reintroduced feedback and manipulated the followers’ response speed so that they initiated their button presses faster in counterimitation than in imitation. If the weak version of the anticipation account is correct, we would expect leaders to show an imitation facilitation effect. However, if leaders simply adapt to the speed with which followers respond, they should now be faster in counterimitation than in imitation.

## Experiment 2

### Methods

We recruited a further 48, previously unacquainted participants (36 female, 12 male; all right-handed with no motor disorders; they formed same-gender pairs). The participants were Edinburgh University students and were paid £6. An additional pair of participants was tested but was excluded from the study prior to data analysis (one participant from that pair reported being left-handed after completing the task). Ethical approval and participants’ consent were obtained as in Experiment 1.

We used the same setup and stimuli as in Experiment 1, although this time participants could see and hear each other; that is, there was no divider, and the leader did not wear earplugs or headphones (Fig. 1, panel b). As previously, we asked the followers to observe the leaders and to produce either the same (imitation) or the opposite (counterimitation) type of press. In addition, followers were now asked to wear headphones (Sony MDR-NC60), and we explained that they would hear some auditory cues. In the imitation block, followers heard a single-tone “GO signal” (160 ms, 800 Hz) played 800–1,075 ms after the onset of the trial. They were instructed to withhold their response until they had heard the tone. In counterimitation, followers heard either a short (80 ms) or a long (240 ms, 800 Hz) single tone, played at trial onset. We told them that the short tone indicated they would need to perform a short press, and conversely, the long tone indicated a long press. The followers were instructed to use these cues to prepare their responses to the upcoming leader’s actions.

The instructions were given to the follower separately so that the leader was unware of the purpose of the auditory cues. However, each participant acted in both roles throughout the experiment. Half of the participants started as leader and then swapped roles, to perform the task again as a follower. Hence, these participants were unaware of the auditory cue instructions while acting as a leader. The other half of the participants started as a follower and then carried on to be leader. This group was therefore aware of the follower’s instructions while they acted as leader. To accommodate this new between-participants factor, we increased the sample size as specified above. Leaders received instructions identical to those in Experiment 1.

### Results

As in Experiment 1, warm-up trials, error trials (3.59 %), and outliers (2.13 %) were removed before analyzing the leader’s responses. Error trials for both participants (18.04 %), as well as further outliers (1.33 %), were excluded for the follower’s analyses.

With regard to the follower’s behavior, we successfully reversed the typical RT pattern (Fig. 2, panel b). Followers were now significantly slower in the imitation than in the counterimitation condition (*M* = 654 vs. 298 ms), *t*(47) = –16.23, *p* < .001, *r* = .92, 95 % CI = [–402, –314], and pairwise comparisons showed that this was the case for both long leader presses (*M* = 533 vs. 286 ms), *t*(47) = –10.45, *p* < .001, *r* = .84, 95 % CI = [–302, –204], and short leader presses (*M* = 781 vs. 310 ms), *t*(47) = –21.06, *p* < .001, *r* = .95, 95 % CI = [–512, –423]. See the supplemental material for the full ANOVA results.

Crucially, we observed the same pattern in leaders’ RTs: Leaders were significantly slower in imitation than in counterimitation (*M* = 470 vs. 450 ms), *t*(47) = –3.06, *p* = .004, *r* = .41, 95 % CI = [–31, –6] (Fig. 2, panel b). Again, this difference was significant both for long (*M* = 481 vs. 462 ms), *t*(47) = –2.78, *p* = .008, *r* = .38, 95 % CI = [–31, –5], and short (*M* = 459 vs. 439 ms), *t*(47) = –2.87, *p* = .006, *r* = .39, 95 % CI = [–33, –6], presses. Furthermore, it was not affected by whether the leaders were aware of the followers’ instructions. A 2 (Condition: imitation vs. counterimitation) × 2 (Leader Press Type: short vs. long) × 2 (Leader Awareness: aware vs. unaware) mixed ANOVA showed that the interaction between condition and leader awareness was not significant [*F*(1, 46) = 2.90, *p* = .095, *η*_G_^2^ < .01]. All other interactions were also nonsignificant (*F*s < 1).

These results may indicate that leaders adapted their response speed to their partners’. To further test this, we calculated the mean difference between the imitation and counterimitation conditions for each participant acting as either follower or leader, and then correlated these differences within participant pairs. There was a positive correlation between the differences for the followers and the leaders within the same pair [*r*(48) = .36, *p* = .011], suggesting that leaders showed larger differences between conditions when their partners did, too. Further corroborating our predictions, we found a similar correlation in a separate experiment that was a direct replication of Pfister et al. (2013) [*r*(24) = .53, *p* = .008; see the supplemental material for details about the replication experiment]. Interestingly, such a correlation was not significant in Experiment 1, in which leaders could not see the followers [*r*(24) = .04, *p* > .250].

Moreover, we investigated whether the leader’s RT on the current trial could be predicted by her partner’s RT on the preceding trial. We ran a linear mixed-effect model with by-participants random intercepts and slopes, and with no correlations between the random effects (the maximal-structure model did not converge). We specified the follower’s RT on the preceding trial as a predictor of the leader’s RT on the current trial, and found that the leader’s action was faster, the faster her partner’s action had been on the preceding trial (*B* = 4.60, *t* = 2.65). This effect was qualified by a significant interaction with condition (*B* = –8.50, *t* = –2.53; Fig. S2, panel b). In counterimitation, we found a positive relationship (*B* = 8.91, *t* = 3.23), indicating local adaptation. In imitation, however, no significant association emerged (*B* = 0.14, *t* = 0.07), most likely because the followers acted in response to a randomly timed GO signal, which rendered adaptation not possible. Finally, a significant association between the follower’s RT on the previous trial and the leader’s RT on the current trial was also present in our replication of Pfister et al. (2013) (*B* = 7.91, *t* = 2.14; Fig. S2, panel c). To the contrary, no such association was apparent in Experiment 1, indicating that local temporal adaptation was not possible without perceptual feedback (*B* = –1.38, *t* = –0.68; Fig. S2, panel a).^2^

## General discussion

Taken together, our findings demonstrate that spontaneous adaptation of response speed, and not high-level anticipation of partners’ actions, is the key mechanism at play in this task. The response facilitation among leaders (Pfister et al., 2013) disappeared once we removed auditory and visual information about their partners’ behavior, suggesting that perceptual feedback was necessary for the emergence of this effect (Exp. 1). Crucially, we showed that the effect among leaders can also be reversed by manipulating the followers’ RT pattern (Exp. 2). When followers responded faster in counterimitation than in imitation, leaders were also faster in the former than in the latter condition. This occurred despite the fact that followers’ responses were still incompatible in counterimitation and compatible in imitation.

Therefore, our results are not consistent with the high-level anticipation account. Leaders’ behavior was influenced by the temporal features of the followers’ responses, and we found no evidence that leaders formed abstract representations of their partners’ actions (i.e., represented those actions as being either short or long). We propose that our findings are better explained by a low-level mechanism of spontaneous temporal adaptation. In support of this claim, the magnitude of the condition difference for the leader was correlated with the magnitude of the condition difference for the follower in the same pair only if the partners could observe each other (i.e., in Exp. 2 and our replication of Pfister et al., 2013, but not in Exp. 1). This is in line with previous studies showing that temporal adaptation is contingent on perceptual information uptake (Nowicki, Prinz, Grosjean, Repp, & Keller, 2013; Richardson, Marsh, & Schmidt, 2005). Moreover, when leaders could observe followers, the leaders’ RT on the current trial was predicted by the followers’ RT on the preceding trial. This indicates that the adaptation occurred locally, on a trial-by-trial basis. Similarly, a recent study revealed that dyads engaged in a joint tapping task showed mutual temporal adaptation on a tap-to-tap basis (Konvalinka et al., 2010).

In light of our findings, we argue for a reconsideration of other phenomena that have traditionally been explained by appealing solely to high-level processes. There is already evidence that agents do not form representations of their partner’s actions when the partner is outside their peripersonal space (Guagnano, Rusconi, & Umiltà, 2010), which is consistent with an important role for perceptual feedback. More importantly, apparent evidence for action co-representation (Sebanz, Knoblich, & Prinz, 2003) can be obtained when the partner is inactive or is replaced with an attention-grabbing object (Dolk, Hommel, Prinz, & Liepelt, 2013). Recent, more parsimonious accounts of joint action have posited that agents do not always need to represent and anticipate each others’ actions (Vesper, Butterfill, Knoblich, & Sebanz, 2010; Wenke et al., 2011). We suggest that low-level mechanisms like temporal adaptation should be considered whenever investigating human coordination (cf. Richardson, Campbell, & Schmidt, 2009; Vesper & Richardson, 2014).

Finally, it is important to note that we do not argue that anticipation plays no role in joint action. Agents flexibly switch between different coordination processes, given the task constraints (e.g., Skewes, Skewes, Michael, & Konvalinka, 2015; Vesper, Schmitz, Safra, Sebanz, & Knoblich, 2016), so anticipation of the coactor’s actions may be involved in some instances of coordination. Moreover, recent accounts of rhythmic joint action suggest that agents anticipate the temporal features of their coactor’s action and that coordination depends on both temporal anticipation and adaptation (Keller, Novembre, & Hove, 2014; Konvalinka et al., 2010; van der Steen & Keller, 2013). Future research should aim to uncover the relationship between anticipation and adaptation, and should further investigate the role of task structure in eliciting different coordination mechanisms. Our results show that it is essential for researchers to consider both the high- and low-level perspectives when building and testing theoretical frameworks of joint action. Only then will these models offer robust explanations and reflect the rich interplay between different mechanisms that shape human coordination.

## Electronic supplementary material

Below is the link to the electronic supplementary material.

ESM 1 (DOCX 146 kb)

## Footnotes

1 Outlier rejection did not affect the results in any of the analyses reported in this article: The same pattern of results was obtained when outliers were retained (see the supplemental material).

2 We found a corresponding pattern of results in an additional cross-correlation analysis: The leader’s RT on the current trial was positively correlated with the follower’s RT on the previous trial in Experiment 2 and in the replication experiment, but not in Experiment 1 (see the supplemental material).

## References

[CR1] Atmaca S, Sebanz N, Knoblich G (2011). The joint flanker effect: Sharing tasks with real and imagined co-actors. Experimental Brain Research.

[CR2] Böckler A, Knoblich G, Sebanz N (2012). Effects of a coactor’s focus of attention on task performance. Journal of Experimental Psychology: Human Perception and Performance.

[CR3] Dolk T, Hommel B, Colzato LS, Schütz-Bosbach S, Prinz W, Liepelt R (2014). The joint Simon effect: A review and theoretical integration. Frontiers in Psychology.

[CR4] Dolk T, Hommel B, Prinz W, Liepelt R (2013). The (not so) social Simon effect: A referential coding account. Journal of Experimental Psychology: Human Perception and Performance.

[CR5] Fowler CA, Richardson MJ, Marsh KL, Shockley KD, Fuchs A, Jirsa VK (2008). Language use, coordination, and the emergence of cooperative action. Coordination: Neural, behavioral and social dynamics.

[CR6] Gambi C, Van de Cavey J, Pickering MJ (2015). Interference in joint picture naming. Journal of Experimental Psychology: Learning, Memory, and Cognition.

[CR7] Goebl W, Palmer C (2009). Synchronization of timing and motion among performing musicians. Music Perception.

[CR8] Guagnano D, Rusconi E, Umiltà CA (2010). Sharing a task or sharing space? On the effect of the confederate in action coding in a detection task. Cognition.

[CR9] Heyes C (2011). Automatic imitation. Psychological Bulletin.

[CR10] Hommel B, Prinz W, Beisert M, Herwig A (2013). Ideomotor action control: On the perceptual grounding of voluntary actions and agents. Action science: Foundations of an emerging discipline.

[CR11] Hommel B, Müsseler J, Aschersleben G, Prinz W (2001). The theory of event coding: A framework for perception and action. Behavioral and Brain Sciences.

[CR12] Jung C, Holländer A, Müller K, Prinz W (2011). Sharing a bimanual task between two: Evidence of temporal alignment in interpersonal coordination. Experimental Brain Research.

[CR13] Keller PE, Morganti F, Carassa A, Riva G (2008). Joint action in music performance. Enacting intersubjectivity: A cognitive and social perspective to the study of interactions.

[CR14] Keller PE, Novembre G, Hove MJ (2014). Rhythm in joint action: Psychological and neurophysiological mechanisms for real-time interpersonal coordination. Philosophical Transactions of the Royal Society B.

[CR15] Knoblich G, Butterfill S, Sebanz N, Ross BH (2011). Psychological research on joint action: Theory and data. The psychology of learning and motivation: Advances in research and theory.

[CR16] Knoblich G, Jordan JS (2003). Action coordination in groups and individuals: Learning anticipatory control. Journal of Experimental Psychology: Learning, Memory, and Cognition.

[CR17] Konvalinka I, Vuust P, Roepstorff A, Frith CD (2010). Follow you, follow me: Continuous mutual prediction and adaptation in joint tapping. Quarterly Journal of Experimental Psychology.

[CR18] Kourtis D, Sebanz N, Knoblich G (2013). Predictive representation of other people’s actions in joint action planning: An EEG study. Social Neuroscience.

[CR19] Kunde W (2001). Response-effect compatibility in manual choice reaction tasks. Journal of Experimental Psychology: Human Perception and Performance.

[CR20] Loehr JD, Vesper C (2016). The sound of you and me: Novices represent shared goals in joint action. Quarterly Journal of Experimental Psychology.

[CR21] Mills PF, van der Steen MC, Schultz BG, Keller PE (2015). Individual differences in temporal anticipation and adaptation during sensorimotor synchronization. Timing and Time Perception.

[CR22] Müller R (2015). Does the anticipation of compatible partner reactions facilitate action planning in joint tasks?. Psychological Research.

[CR23] Neda Z, Ravasz E, Brechte Y, Vicsek T, Barabasi A-L (2000). The sound of many hands clapping. Nature.

[CR24] Nowicki L, Prinz W, Grosjean M, Repp BH, Keller PE (2013). Mutual adaptive timing in interpersonal action coordination. Psychomusicology: Music, Mind, and Brain.

[CR25] Pfister R, Dignath D, Hommel B, Kunde W (2013). It takes two to imitate: Anticipation and imitation in social interaction. Psychological Science.

[CR26] Pfister R, Dolk T, Prinz W, Kunde W (2014). Joint response–effect compatibility. Psychonomic Bulletin & Review.

[CR27] Pfister R, Pfeuffer CU, Kunde W (2014). Perceiving by proxy: Effect-based action control with unperceivable effects. Cognition.

[CR28] Repp BH (2005). Sensorimotor synchronization: A review of the tapping literature. Psychonomic Bulletin & Review.

[CR29] Repp BH, Su YH (2013). Sensorimotor synchronization: A review of recent research (2006–2012). Psychonomic Bulletin & Review.

[CR30] Richardson MJ, Campbell WL, Schmidt RC (2009). Movement interference during action observation as emergent coordination. Neuroscience Letters.

[CR31] Richardson MJ, Marsh KL, Isenhower R, Goodman J, Schmidt RC (2007). Rocking together: Dynamics of intentional and unintentional interpersonal coordination. Human Movement Science.

[CR32] Richardson MJ, Marsh KL, Schmidt RC (2005). Effects of visual and verbal interaction on unintentional interpersonal coordination. Journal of Experimental Psychology: Human Perception and Performance.

[CR33] Ryskin RA, Benjamin AS, Tullis J, Brown-Schmidt S (2015). Perspective-taking in comprehension, production, and memory: An individual differences approach. Journal of Experimental Psychology: General.

[CR34] Schmidt RC, Fitzpatrick P, Caron R, Mergeche J (2011). Understanding social motor coordination. Human Movement Science.

[CR35] Sebanz N, Bekkering H, Knoblich G (2006). Joint action: Bodies and minds moving together. Trends in Cognitive Sciences.

[CR36] Sebanz N, Knoblich G, Prinz W (2003). Representing others’ actions: Just like one’s own?. Cognition.

[CR37] Shintel H, Keysar B (2009). Less is more: A minimalist account of joint action in communication. Topics in Cognitive Science.

[CR38] Skewes JC, Skewes L, Michael J, Konvalinka I (2015). Synchronised and complementary coordination mechanisms in an asymmetric joint aiming task. Experimental Brain Research.

[CR39] van der Steen MM, Keller PE (2013). The ADaptation and Anticipation Model (ADAM) of sensorimotor synchronization. Frontiers in Human Neuroscience.

[CR40] van der Wel RPRD, Sebanz N, Knoblich G (2014). Do people automatically track others’ beliefs? Evidence from a continuous measure. Cognition.

[CR41] Vesper C, Butterfill S, Knoblich G, Sebanz N (2010). A minimal architecture for joint action. Neural Networks.

[CR42] Vesper C, Richardson MJ (2014). Strategic communication and behavioral coupling in asymmetric joint action. Experimental Brain Research.

[CR43] Vesper C, Schmitz L, Safra L, Sebanz N, Knoblich G (2016). The role of shared visual information for joint action coordination. Cognition.

[CR44] Wenke D, Atmaca S, Holländer A, Liepelt R, Baess P, Prinz W (2011). What is shared in joint action? Issues of co-representation, response conflict, and agent identification. Review of Philosophy and Psychology.

