# Supplementary material for: Spontaneous adaptation explains why people act faster when being imitated
Source: Psychon Bull Rev. 2016 Aug 16;24(3):842–8. doi: 10.3758/s13423-016-1141-3 (PMC5486872; doi:10.3758/s13423-016-1141-3)
Supplement: Supplementary file 1 — (DOCX 146 kb) [file 13423_2016_1141_MOESM1_ESM.docx]

# Replication experiment

**Methods**

Based on the sample size chosen by Pfister et al. (2013), we invited 24 previously unacquainted participants (18 female, 6 male; right-handed; same-gender pairs). Participants were Edinburgh University students with no motor disorders and were paid £6. The study was approved by the local ethics committee and consent was obtained from all participants.

We used the same procedure and materials as in our Experiment 1. However, similarly to the original study (Pfister et al., 2013), there was no divider and participants could see and hear each other while doing the task (Fig. 2).

# Results

Warm-up trials, error trials (4.31%) and outliers (2.25%) were removed prior to analysing the leader’s responses. Error trials for both participants (8.65%), as well as further outliers (1.45%) were removed prior to the follower’s analyses.

We found that followers’ responses were facilitated in the imitation condition (Fig. S1, panel *c*): Followers were significantly faster in imitation than counter-imitation (*M* = 325 ms vs *M* = 447 ms; *t*(23) = 6.71, *p* < .001, *r* = .81, 95% CI = [84, 159]). In follow-up pairwise comparisons, we then tested whether this effect was present for both long and short key presses. After excluding one follower who showed an extremely high error rate (48.94%) for long presses in imitation, we found that the imitation facilitation effect was present for both long (*M* = 307 ms vs *M* = 370 ms; *t*(22) = 3.54, *p* = .002, *r* = .60, 95% CI = [26, 100]) and short leader presses (*M* = 333 ms vs *M* = 495 ms; *t*(22) = 6.20, *p* < .001, *r* = .80, 95% CI = [108, 216]).

Importantly, we replicated the original finding that leaders were faster in imitation than counter-imitation (*M* = 415 ms vs *M* = 436 ms; *t*(23) = 3.22, *p* = .004, *r* = .56, 95% CI = [8, 37]) (Fig. S1, panel *c*). Pairwise comparisons showed this difference was significant both for long (*M* = 426 ms vs *M* = 444 ms; *t*(23) = 2.46, *p* = .022, *r* = .46, 95% CI = [3, 33]) and short presses (*M* = 405 ms vs *M* = 427 ms; *t*(23) = 3.24, *p* = .004, *r* = .56, 95% CI = [9, 39]).

**a**


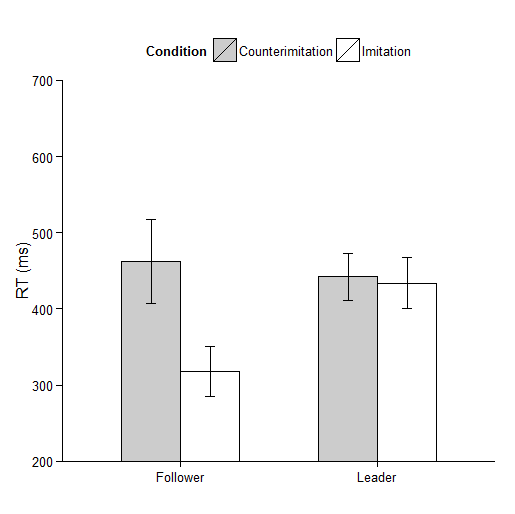


**b**

**
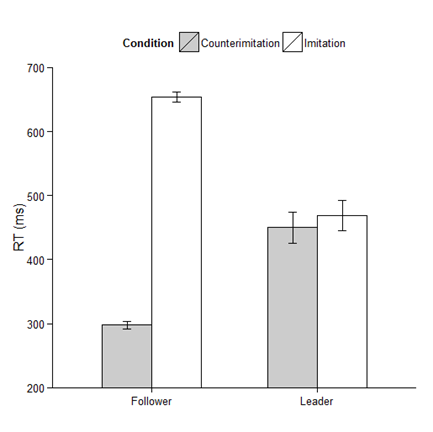
**

**c**

#
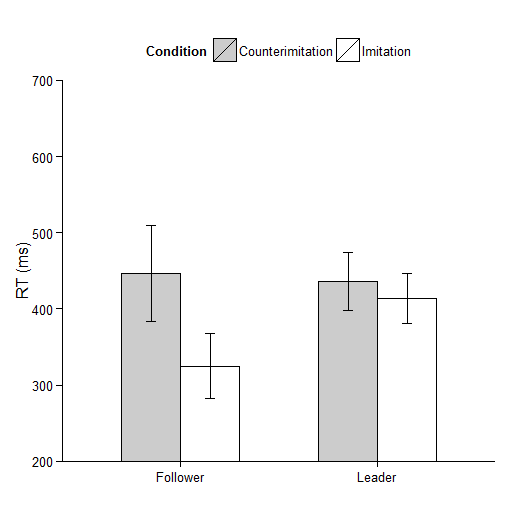


**Fig. S1.** Mean leaders’ and followers’ RT in imitation and counter-imitation, in Experiment 1, Experiment 2 and Replication. Error bars represent 95% CI. Panel *a* shows Experiment 1, panel *b* Experiment 2, and panel *c* Replication. Panels *a* and *b* correspond to Fig. 3 and 4 (respectively) in the main paper.

# Error rate

# Experiment 1

A 2 (Condition) X 2 (Leader’s Press Type) within-subjects ANOVA on arcsine-transformed percentage scores showed that leader’s error trials were equally distributed between conditions and press types, (Leader’s Press Type: *F*(1,23) = 1.42, *p* = .245, $\eta_{G}^{2}$ = .03; Condition: *F*(1,23) = .38, *p* > .250, $\eta_{G}^{2}$ < .01; interaction: *F*(1,23) = .40, *p* > .250, $\eta_{G}^{2}$ < .01). Similarly, there were no significant effects on followers’ error rate (Leader’s Press Type: *F*(1,23) = 1.15, *p* > .250, $\eta_{G}^{2}$ < .01; Condition: *F*(1,23) = 0.01, *p* > .250, $\eta_{G}^{2}$ < .01; interaction: *F*(1,23) < .001, *p* > .250, $\eta_{G}^{2}$ < .01).

# Experiment 2

We found no significant effects on leaders’ error rate (Leader’s Press Type: *F*(1,47) = 3.45, *p* = .069, $\eta_{G}^{2}$ = .03; Condition: *F*(1,47) = .06, *p* > .250, $\eta_{G}^{2}$ < .01; interaction: *F*(1,47) = .94, *p* > .250, $\eta_{G}^{2}$ < .01). However, there was a significant main effect of condition on followers’ error rate, indicating that followers made more errors in counter-imitation than imitation (16.97% vs 11.94%; *F*(1,47) = 5.69, *p* = .021, $\eta_{G}^{2}$ = .02). No other effects were significant (Leader’s Press Type: *F*(1,47) = .22, *p* > .250, $\eta_{G}^{2}$ < .01; interaction *F*(1,47) = .73, *p* > .250, $\eta_{G}^{2}$ < .01).

A possible worry is that leaders might have been affected by the followers’ error rate in counter-imitation. There are two reasons to dismiss this possibility: (a) leaders did not show a corresponding difference in the distribution of error rates and (b) had leaders experienced difficulties due to followers’ high error rate in counter-imitation, they would have slowed down; instead, they sped up in counter-imitation, adapting to followers’ RT.

# Replication experiment

Leaders’ error trials were equally distributed between conditions and press types (Leader’s Press Type: *F*(1,23) = .15, *p* > .250, $\eta_{G}^{2}$ < .01; Condition: *F*(1,23) = .16, *p* > .250, $\eta_{G}^{2}$ < .01; interaction: *F*(1,23) = .54, *p* > .250, $\eta_{G}^{2}$ < .01). We found no significant effects for followers’ error rate (Leader’s Press Type: *F*(1,23) = .03, *p* > .250, $\eta_{G}^{2}$ < .01; Condition: *F*(1,23) = 0.12, *p* > .250, $\eta_{G}^{2}$ < .01; interaction: *F*(1,23) = .16, *p* > .250, $\eta_{G}^{2}$ < .01).

**Additional analyses (ANOVA)**

**Experiment 1**

**Tab. S1**

| Experiment 1: Followers’ and leaders’ average response times (RT) by Leader’s Press Type and Condition | | | | |
| --- | --- | --- | --- | --- |
|  | Followers’ RT | | Leaders’ RT | |
| Condition | Leader’s Press Type | | Leader’s Press Type | |
|  | short | long | short | long |
| imitation | 328 | 308 | 425 | 444 |
| counter-imitation | 526 | 392 | 440 | 443 |
| *Note*. Average RT were calculated for data excluding followers’ and leaders’ error trials and outliers (see the main paper for details). Response times are reported in *ms*. | | | | |

We ran a 2 (Condition: imitation vs counter-imitation) X 2 (Leader’s Press Type: short vs long) within-subjects ANOVA on followers’ RT. The analysis revealed main effects of Condition (*F*(1,23) = 34.21, *p* < .001, $\eta_{G}^{2}$ = .27) and Leader’s Press Type (*F*(1,23) = 12.02, *p* = .002, $\eta_{G}^{2}$ = .07), qualified by a significant interaction (*F*(1,23) = 14.42, *p* < .001, $\eta_{G}^{2}$ = .05). Followers were faster in imitation than counter-imitation, and the difference between conditions was bigger for short than for long presses (Tab. S1).

We conducted a similar analysis on leaders’ RT and found an interaction between Condition and Leader’s Press Type (*F*(1,23) = 5.67, *p* = .026, $\eta_{G}^{2}$ < .01). Importantly, pairwise comparisons showed that this interaction was not driven by the effect of Condition (see the tests reported in the main paper). Instead, it appeared to be driven by a significant difference in leaders’ RT between short and long presses in the imitation condition (*t*(23) = 2.92, *p* = .008, *r* = .52, 95% CI = [6, 34]; Tab. S1). There was no such difference in counter-imitation (*t*(23) = 0.47, *p* > .250, *r* = .10, 95% CI = [-14, 23]). We found no main effects of Condition (*F*(1,23) = 1.32, *p* > .250, $\eta_{G}^{2}$ < .01) or Leader’s Press Type (*F*(1,23) = 2.77, *p* = .109, $\eta_{G}^{2}$ < .01).

**Experiment 2**

**Tab. S2**

| Experiment 2: Followers’ and leaders’ average response times (RT) by Leader’s Press Type and Condition | | | | |
| --- | --- | --- | --- | --- |
|  | Followers’ RT | | Leaders’ RT | |
| Condition | Leader’s Press Type | | Leader’s Press Type | |
|  | short | long | short | long |
| imitation | 781 | 533 | 459 | 481 |
| counter-imitation | 310 | 286 | 439 | 462 |
| *Note*. Average RT were calculated for data excluding followers’ and leaders’ error trials and outliers (see the main paper for details). Response times are reported in *ms*. | | | | |

A 2 (Condition: imitation vs counter-imitation) X 2 (Leader’s Press Type: short vs long) within-subjects ANOVA on followers’ RT found a main effect of Condition (*F*(1,47) = 298.11, *p* < .001, $\eta_{G}^{2}$ = .61) and a main effect of Leader’s Press Type (*F*(1,47) = 132.57, *p* < .001, $\eta_{G}^{2}$ = .17). Furthermore, there was a significant interaction suggesting that followers were slower in imitation as compared to counter-imitation, and that this difference was greater for short than for long leader presses (*F*(1,47) = 110.64, *p* < .001, $\eta_{G}^{2}$ = .12; Tab. S2).

With regards to leaders’ RT, the analysis confirmed that leaders were slower in the imitation than in the counter-imitation condition (*F*(1,47) = 8.96, *p* = .004, $\eta_{G}^{2}$ = .01). Moreover, there was a main effect of Leader’s Press Type on leaders’ RT, suggesting that leaders responded faster for short than long presses (*F*(1,47) = 21.70, *p* < .001, $\eta_{G}^{2}$ = .02). The interaction was not significant (*F*(1,47) = 0.10, *p* > .250, $\eta_{G}^{2}$ < .01).

**Replication experiment**

**Tab. S3**

| Replication experiment: Followers’ and leaders’ average response times (RT) by Leader’s Press Type and Condition | | | | |
| --- | --- | --- | --- | --- |
|  | Followers’ RT | | Leaders’ RT | |
| Condition | Leader’s Press Type | | Leader’s Press Type | |
|  | short | long | short | long |
| imitation | 332 | 311 | 405 | 426 |
| counter-imitation | 485 | 361 | 427 | 444 |
| *Note*. Average RT were calculated for data excluding followers’ and leaders’ error trials and outliers (see the main paper for details). In addition, followers’ RT were calculated on data excluding one participant who showed an extremely high error rate. Response times are reported in *ms*. | | | | |

The analysis on followers’ RT revealed main effects of Condition (*F*(1,22) = 42.26, *p* < .001, $\eta_{G}^{2}$ = .16) and Leader’s Press Type (*F*(1,22) = 11.84, *p* = .002, $\eta_{G}^{2}$ = .08), qualified by a significant interaction (*F*(1,22) = 12.30, *p* = .002, $\eta_{G}^{2}$ = .03). Followers were faster in imitation than counter-imitation, and the difference between conditions was bigger for short than for long presses (Tab. S3).^[[1]](#footnote-1)^

Leaders’ RT were shorter in imitation than counter-imitation condition (*F*(1,23) = 9.35, *p* = .006, $\eta_{G}^{2}$ = .01) and shorter for short than long leader presses (*F*(1,23) = 7.62, *p* = .011, $\eta_{G}^{2}$ = .01; Tab. S3). The interaction between Condition and Leader’s Press Type was not significant (*F*(1,23) = 1.36, *p* = .250, $\eta_{G}^{2}$ < .01).

**Cross-correlation analysis**

In order to further investigate the relationship between the leader’s RT on the current trial and the follower’s RT on the preceding trial, we conducted a cross-correlation analysis (maximum lag of 1) on the data from all three experiments. We assumed that the time-series of participant responses obtained in our task had local stationarity and performed the analysis in non-overlapping windows of 6 observations (cf. Konvalinka et al., 2010).

For each dyad, we calculated average cross-correlation coefficient for lag = -1 (estimates a correlation between the leader’s RT on the current trial and the follower’s RT on the preceding trial), lag = 0 (leader’s RT on the current trial and the follower’s RT on the current trial) and lag = 1 (leader’s RT on the current trial and the follower’s RT on the following trial). Average coefficients were then transformed into Fischer Z-scores and entered into a one-way MANOVA with Experiment as a between-subjects factor (Experiment: Experiment 1 vs Experiment 2 vs Replication) and average coefficients for lags -1, 0, and 1 as the dependent variables.

**Tab. S4**

| Experiments 1-2 and Replication experiment: Average Z-transformed cross-correlation coefficients for lags -1, 0, and 1 | | | |
| --- | --- | --- | --- |
|  | Lag | | |
|  | -1 | 0 | 1 |
| Experiment 1 | 0.003 | -0.083 | 0.023 |
| Experiment 2 | 0.057 | -0.259 | 0.040 |
| Replication | 0.064 | -0.084 | 0.022 |

If the condition effect on leaders’ RT is due to temporal anticipation (i.e., leaders anticipate followers’ response speed on the current trial), we would expect to see a positive correlation for lag 0. Instead, we observed negative correlations for lag 0 in all experiments, suggesting that temporal anticipation did not affect leader’s action execution on the current trial (Tab. S4). In addition, the correlation was stronger in Experiment 2 than in Experiment 1 and the Replication experiment, as indicated by the MANOVA (main effect of Experiment: *F*(2,45) = 9.68, *p* < .001) and follow-up pairwise comparisons (Experiment 2 vs 1: *t*(23.35) = 3.48, *p* = .002, *d* = .87, 95% CI = [.07, 28]; Experiment 2 vs Replication: *t*(28.53) = 3.97, *p* < .001, *d* = .99, 95% CI = [.08, 26]; Experiment 1 vs Replication: *t*(20.93) = .01, *p* > .250, *d* = .00, 95% CI = [-.11, .11]). A strong negative correlation in Experiment 2 is likely to be a consequence of the fact that the followers responded to the auditory cue and not directly to leaders’ actions. However, a negative relationship in Experiment 1 and in the Replication experiment could suggest that followers dynamically adapted to leader’s RT (e.g., if the leader was particular slow on a given trial, the follower could compensate by speeding up their own response).^[[2]](#footnote-2)^

If leaders adapt to followers’ RT on the preceding trial, we would expect a positive correlation for lag -1, but only in the experiments where participants could observe each other. Consistently with this prediction, there was a weak positive correlation in Experiment 2 and the Replication experiment, but a null correlation in Experiment 1 (Tab. S4). This numerical trend was reflected by a main effect of Experiment in MANOVA (*F*(2,45) = 3.74, *p* = .031). Furthermore, follow-up pairwise comparisons revealed that Experiment 2 differed significantly from Experiment 1 in terms of correlation strength for lag -1 (*t*(22.93) = -2.95, *p* = .007, *d* = .74, 95% CI = [-.09, -.02]. Similarly, there was a difference between the Replication experiment and Experiment 1, however it was only marginally significant (*t*(17.98) = -2.12, *p* = .048, *d* = .61, 95% CI = [-.12, .00]). Experiment 2 and Replication did not differ (*t*(15.41) = 0.25, *p* > .250, *d* = .06, 95% CI = [-.05, .06]). We found no effect of Experiment with regards to correlation coefficients for lag 1 (*F*(2,45) =.34, *p* > .250).

# Trial-by-trial relationship between follower’s and leader’s RT

**a**


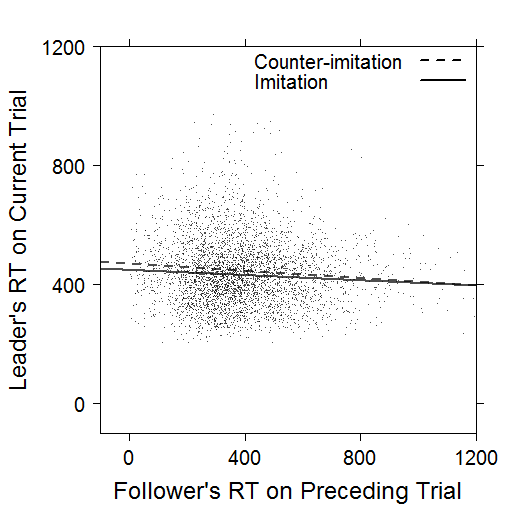


**b**


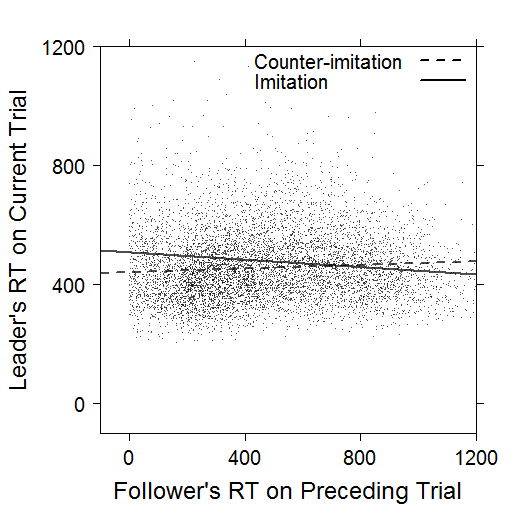


**c**

#
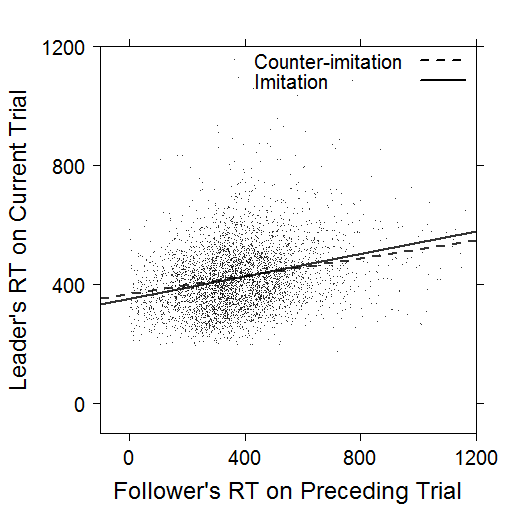


**Fig. S2.** Relationship between Follower’s RT on preceding trial and Leader’s RT on current trial. Lines represent regression lines for imitation (solid) and counter-imitation (dashed). Panel *a* shows Experiment 1, panel *b* Experiment 2, and panel *c* Replication experiment.

**Main analyses on data including outliers**

We checked whether outlier rejection affected the key results in Experiments 1-2 and the Replication experiment. For each experiment, we ran the analyses on the data excluding the leader and follower error trials, but retaining the outliers.

**Experiment 1**

We obtained the same pattern of results as in the analyses on the data without outliers. Followers were significantly faster in imitation than counter-imitation (*M* = 188 ms vs *M* = 384 ms; *t*(23) = 6.17, *p* < .001, *r* = .79, 95% CI = [139, 279]), and this effect was present both for long (*M* = 46 ms vs *M* = 235 ms; *t*(23) = 4.22, *p* < .001, *r* = .66, 95% CI = [94, 275]) and short leader presses (*M* = 333 ms vs *M* = 536 ms; *t*(23) = 7.70, *p* < .001, *r* = .85, 95% CI = [152, 264]). Leaders showed no difference in response speed between imitation and counter-imitation (*M* = 442 ms vs *M* = 449 ms; *t*(23) = 1.07, *p* > .250, *r* = .22, 95% CI = [-6, 19]). Follow-up comparisons showed there was no difference between conditions both for long (*M* = 453 ms vs *M* = 452 ms; *t*(23) = -.03, *p* > .250, *r* = .01, 95% CI =[-13, 13]) and short presses (*M* = 431 ms vs *M* = 445 ms; *t*(23) = 1.61, *p* = .121, *r* = .32, 95% CI = [-4, 30]).

**Experiment 2**

Again, we obtained the same pattern of results as for the data without outliers. Followers responded slower in imitation than counter-imitation (*M* = 657 ms vs *M* = 307 ms; *t*(47) = -16.10, *p* < .001, *r* = .92, 95% CI = [-397, -309]), both for long (*M* = 531 ms vs *M* = 290 ms; *t*(47) = -10.24, *p* < .001, *r* = .83, 95% CI = [-294, -198]) and short leader presses (*M* = 788 ms vs *M* = 322 ms; *t*(47) = -21.13, *p* < .001, *r* = .95, 95% CI = [-509,-420]). Similarly, leaders were significantly slower in imitation than counter-imitation (*M* = 478 ms vs *M* = 458 ms; *t*(47) = -3.11, *p* = .003, *r* = .41, 95% CI = [-32, -7]), and this difference was significant both for long (*M* = 490 ms vs *M* = 470 ms; *t*(47) = -3.01, *p* = .004, *r* = .40, 95% CI = [-32, -6]) and short presses (*M* = 467 ms vs *M* = 446 ms; *t*(47) = -2.89, *p* = .006, *r* = .39, 95% CI = [-34,-6]).

**Replication experiment**

As in the analyses on the data without outliers, followers were faster in imitation than counter-imitation (*M* = 331 ms vs *M* = 439 ms; *t*(23) = 6.65, *p* < .001, *r* = .81, 95% CI = [83, 158]), and this effect was significant both for long (*M* = 315 ms vs *M* = 364 ms; *t*(22) = 3.28, *p* = .003, *r* = .57, 95% CI = [22, 100]) and short leader presses (*M* = 339 ms vs *M* = 490 ms; *t*(22) = 6.39, *p* < .001, *r* = .81, 95% CI = [109, 213]).^[[3]](#footnote-3)^

Consistently with the analyses on the data without outliers, leaders were significantly faster in imitation than counter-imitation (*M* = 421 ms vs *M* = 446 ms; *t*(23) = 3.54, *p* = .002, *r* = .59, 95% CI = [10, 39]). However, pairwise comparisons found that this effect was significant only for short presses (*M* = 411 ms vs *M* = 439 ms; *t*(23) = 3.45, *p* = .002, *r* = .58, 95% CI = [-34, -6]), but not for long presses (*M* = 435 ms vs *M* = 452 ms; *t*(23) = 2.05, *p* = .052, *r* = .39, 95% CI = [-.12, 31]).

1. Prior to ANOVA on the follower’s RT’s, we removed data from one participant due to an extremely high error rate (see Results from the Replication experiment). [↑](#footnote-ref-1)
2. Note that this argument is purely speculative, as our experiments were not designed to test adaptation on the side of the follower. [↑](#footnote-ref-2)
3. Prior to the pairwise comparisons for the follower’s RT’s, we removed data from the participant who had extremely high error rate. [↑](#footnote-ref-3)
